# Accurate classification of fresh and charred grape seeds to the varietal level, using machine learning based classification method

**DOI:** 10.1038/s41598-021-92559-4

**Published:** 2021-06-30

**Authors:** Vlad Landa, Yekaterina Shapira, Michal David, Avshalom Karasik, Ehud Weiss, Yuval Reuveni, Elyashiv Drori

**Affiliations:** 1 Department of Computer Science, Ariel University, 40700 Ariel, Israel; 2 Department of Chemical Engineering, Biotechnology and Materials, Ariel University, 40700 Ariel, Israel; 3 Archaeobotanical Laboratory and National Natural History Collection of Plants’ Seeds and Fruits, Institute of Archaeology, Martin (Szusz) Department of Land of Israel Studies and Archaeology, Bar-Ilan University, 5290002 Ramat-Gan, Israel; 4 The National Laboratory for Digital Documentation and Research in Archaeology, Israel Antiquities Authority, Jerusalem, Israel; 5 Department of Physics, Faculty of Natural Sciences, Ariel University, Science Park, 40700 Ariel, Israel; 6 Remote Sensing Lab, Eastern R&D Center, 40700 Ariel, Israel; 7 The Wine Research Center, Eastern Regional R&D Center, 40700 Ariel, Israel

**Keywords:** Classification and taxonomy, Image processing, Machine learning

## Abstract

Grapevine (*Vitis vinifera* L.) currently includes thousands of cultivars. Discrimination between these varieties, historically done by ampelography, is done in recent decades mostly by genetic analysis. However, when aiming to identify archaeobotanical remains, which are mostly charred with extremely low genomic preservation, the application of the genomic approach is rarely successful. As a result, variety-level identification of most grape remains is currently prevented. Because grape pips are highly polymorphic, several attempts were made to utilize their morphological diversity as a classification tool, mostly using 2D image analysis technics. Here, we present a highly accurate varietal classification tool using an innovative and accessible 3D seed scanning approach. The suggested classification methodology is machine-learning-based, applied with the Iterative Closest Point (ICP) registration algorithm and the Linear Discriminant Analysis (LDA) technique. This methodology achieved classification results of 91% to 93% accuracy in average when trained by fresh or charred seeds to test fresh or charred seeds, respectively. We show that when classifying 8 groups, enhanced accuracy levels can be achieved using a "tournament" approach. Future development of this new methodology can lead to an effective seed classification tool, significantly improving the fields of archaeobotany, as well as general taxonomy.

## Introduction

Grapevine is one of the classical fruits of the Old World and an essential part of the oldest group of fruit trees around which horticulture evolved at the Mediterranean basin^1^. This species includes thousands of known cultivars, grown at a wide array of climatic conditions, as well as its wild progenitor (*Vitis vinifera* ssp. *sylvestris*)^2^. Discrimination between grape varieties has been done traditionally using ampelography^3^, a field of classification by the shape and color of leaves, bunches and berries. In recent decades, grape variety identification dramatically evolved, exploiting the development of DNA analysis methods by AFLP^4,5^, SSR^6,7^, and SNPs^8–10^. These techniques are very straightforward and accurate when fresh plant material is available.

In archaeobotany (aka paleoethnobotany), the scientific study of plant remains from archaeological sites for reconstructing and interpreting past environments and human–plant relationships single-species identification is fundamental. Although it may involve much time and great effort, it is of utmost importance as meaningful interpretations and reconstructing reliant on well-identified species^11^. However, this goal is not always achieved, and this bottleneck hampers the researcher’s ability to answer fundamental research questions. One of the main reasons for the mentioned bottleneck is the fact that high genomic preservation is typically found in rare desiccated or waterlogged plant remains^12^. At the same time, most archaeobotanical assemblages worldwide went through the charring process—which badly influences genomic preservation^13,14^. Current methods are limited in producing high quality and quantity of aDNA from charred seeds due to low endogenous DNA content, short DNA fragments with high rates of nucleotide damage, and high rates of modern DNA contamination, leading to the yield of insufficiently reliable genetic data^15^. These facts pose a high barrier preventing the identification of most grape remains in current archeological repositories. Therefore, seeking to identify grape varieties in archaeobotanical grape remains, a different approach is urgently needed.

Grape pips are highly polymorphic^2^. Exploiting this fact, several attempts were reported recently utilizing the diversity in fresh grape pip morphology as a diagnostic tool, using image analysis techniques, aiming to utilize these methods for the identification of fresh and archaeological specimens. First reports in this field appeared in the last decade—in the work of Terral et al*.*^16,17^, where geometrical analysis (using elliptic Fourier transform method) was applied to analyze the 2D outlines of fresh grapevine pips. Using this methodology, a morphological key was created for pips of approximately fifty French grapevine varieties. Also, a significant correlation between pip morphology and the taxonomic relationship was demonstrated. Furthermore, an innovative approach for the investigation of archaeological remains by combined 2D morphometric and genetic methods was recently developed, showing promising results in melon seeds^18,19^.

Over the last decade, 3D scanning technology has advanced dramatically. Besides its significant role in the industry, modern academic studies harnessed this technology to explore and investigate new questions that were never accessible without the 3D acquired information. Various identification techniques were developed, combining the potential of highly accurate 3D scanning and imagery technology, with mathematical and statistical classification methods and innovative tools from the field of computer sciences^20–24^. Furthermore, current developments in cloud-based big-data technologies enable data-driven solutions, applied with increasing numbers of scientific computing studies^25,26^. Machine learning (ML) is undoubtedly the most common data-driven solution approach which finds complex mathematical patterns and relationships inside the data and uses them to bring considerable datasets to the surface^27,28^. Commonly, ML techniques consist of two learning types: supervised learning and unsupervised learning. Supervised learning means that every trained data sample has a known label. The ML model outcome can be categorical or continuous, depending on the nature of the problem. A categorical output can be laid in a simple 1/0 label, and its methodical term is referred to as binary classification. In the case of a continuous outcome, the methodical term is referred to as regression. The algorithms that are used to tackle classification and regression problems include linear regression, Random Forest (RF), decision trees, Support Vector Machines (SVM)^29^, and Linear Discriminant Analysis (LDA)^30^. On the other hand, Unsupervised Learning aims to deduce hidden internal data features and patterns without the need for assigned labels. This type of learning is commonly used for hidden features-based clustering. ML algorithms such as K-Nearest Neighbors (KNN), Principal Component Analysis (PCA), and Self Organizing Map (SOM) are all examples of Unsupervised Learning models^31^. Therefore, using multivariate data analysis methods, adopted from the ML^32^ discipline for classifying agricultural, as well as archeological geometrical^33–35^ and structural features, can be extremely valuable^36,37^.

In a previous publication^38^ we described our efforts in developing a 3D tool for grape variety identification by grape pip structure. It was clearly demonstrated that the 3D method described is a promising tool for grape variety identification using fresh grape pips, as it enabled the separation between different *Vitis vinifera* varieties with high statistical certainty. This method made use of a set of planar curves extracted from a full 3D scan of the seed, which represents its key features. The novelty of this method lies in the combination of scanning methods and the right selection of Fourier coefficients and their weights.

Here, we present an accurate method for varietal classification of charred grape seeds, using an innovative and accessible 3D scanning method, combined with a machine-learning-based classification technique which yields promising results compared with other tested techniques, such as PCA, SVM and KNN, using the complete set of 3D imagery data. This innovative data representation approach introduces additional dimensions for alignment, similarity and features, compare with previous 2D methods. Additionally, it holds the exact morphology data of the scanned object. We also suggest an innovative way of upscaling the analysis to a broader set of varieties. This breakthrough is the first step in developing a computerized classification tool for the identification of grape, and possibly other species of archaeobotanical seeds, at the variety level.

## Experiments and results

### Visualization of grapevine pips

**Figure 1:**
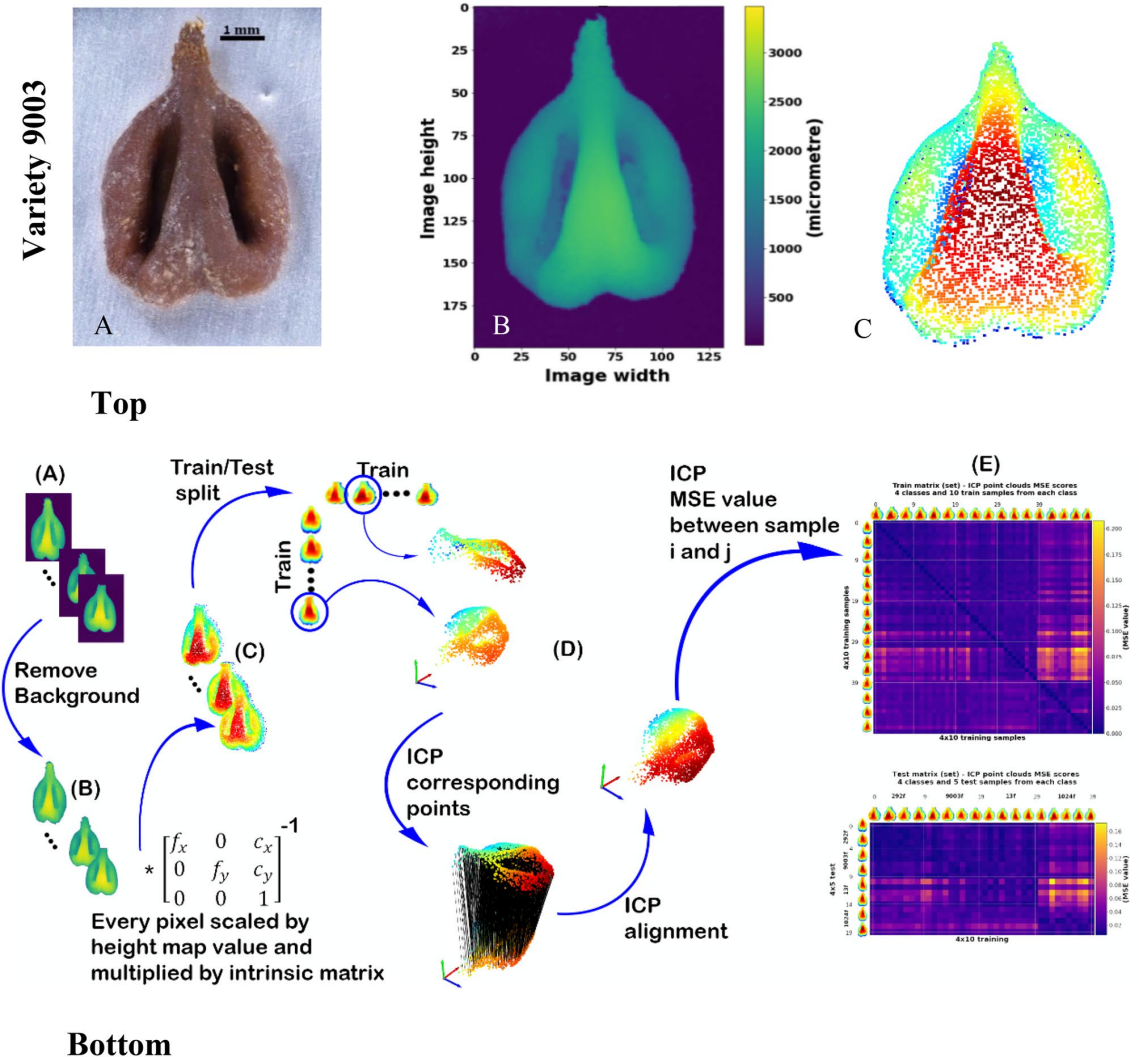
The procedure used for grape pip 3D data acquisition and Train and Test matrix preparation for LDA. (**A**) (Top)—high-quality focused image of grape pip; (**B**) (Top, Bottom)—height map image scan, and (**C**) (Top, Bottom)—3D points cloud representation of fresh pips of variety 9003 (Israeli endogenous variety). (**D**) (Bottom)—Process of applying ICP algorithm to discriminate between two sets of 3D points clouds samples. (**E**) (Bottom)—Training and Test matrices capturing the MSE between two points clouds sets, and serves as input for LDA.

A set of height maps grape seed scans were used for evaluating the performance of the alternative 3D classification method. We selected pips of four grape varieties for scanning, followed by classification of the pips to their initial classes (varieties). Cabernet Sauvignon (N = 15) is a highly esteemed international variety. The three other varieties: ‘292’ (N = 15), ‘13’ (N = 15), and ‘9003’ (N = 15) are *Vitis vinifera* ssp*. sativa* lines that were collected from the Israeli endogenous grape varieties collection in Ariel^39,40^. Figure 1A—top presents high-quality focused image of grape pip. The height map image scan was then converted into a 3D points cloud representation as follows: (1) Every pixel in the height map scan is transformed into a 3D vector representation (${[x*z,y*z,z]}^{T}$), with true scale (Fig. 1B—bottom) and multiplied by the inverse intrinsic matrix (consisted of the focal-lengths, sensors center coordinates in pixels, and skew parameter). Thus, the entire height map representation constitutes a point cloud of that particular scan (Fig. 1C—bottom). (2) Once the point cloud representation is obtained for every seed, we construct a matrix representing similarity “scores” between the two sets of point clouds (Fig. 1D—bottom). Each entry in the matrix represents the similarity between two point clouds. (3) Then, the similarity matrix is used as an input for the LDA (Fig. 1E—bottom). A detailed explanation is described in the materials and methods section; also see (Fig. 1B,C).

### Linear discriminant analysis (LDA) of fresh grape pips

For the first analysis, we evaluated the effectiveness of our classification method with one hundred iteration, utilizing fresh (uncharred) pips from 4 grape varieties (classes). For each iteration, ten out of fifteen pips, from each variety, were randomly chosen as a training set (40 in total), and the remaining five were selected as a test set (20 in total).

**Figure 2:**
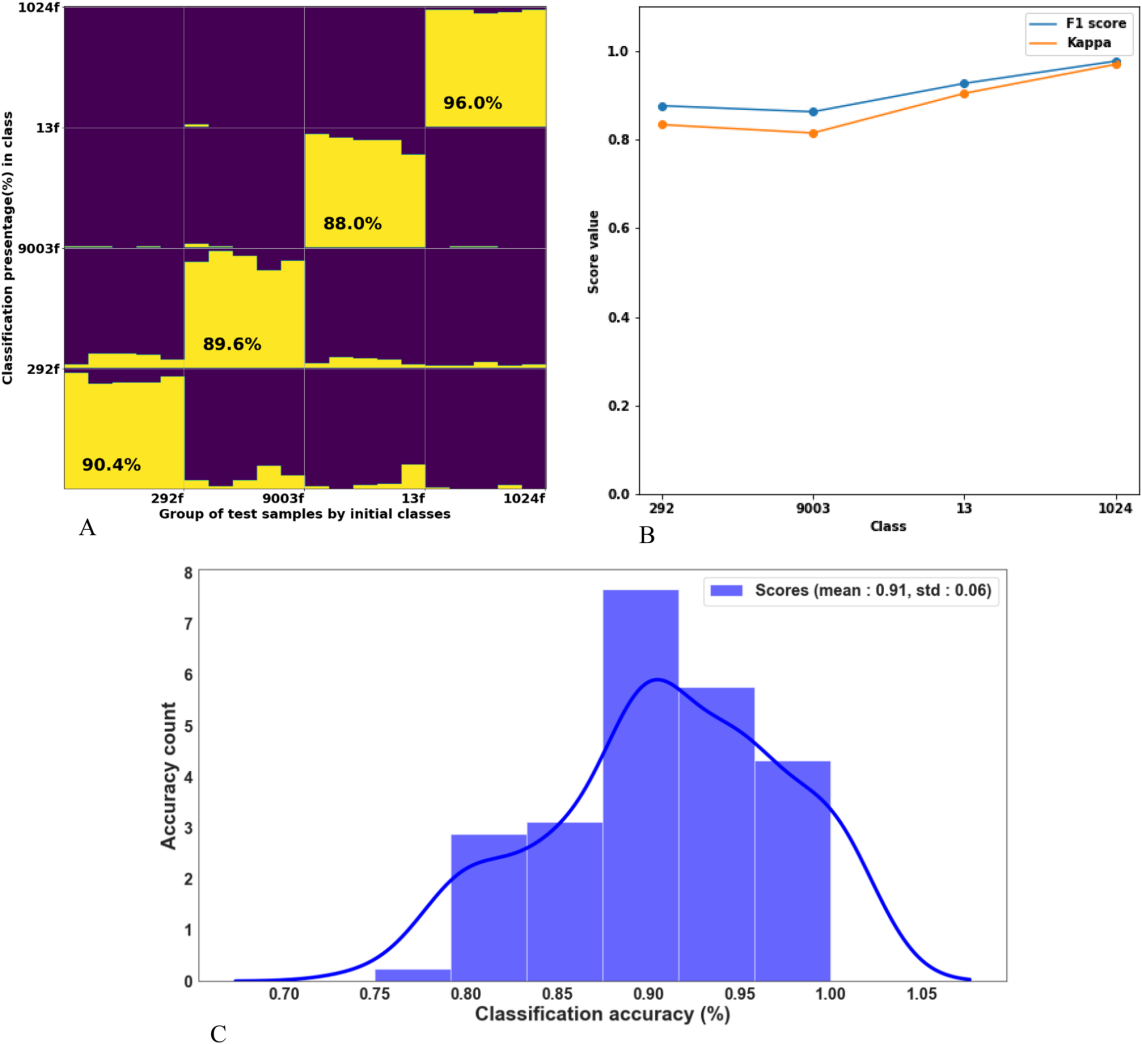
Classification of fresh pips, using a random train set of fresh pips. (**A**) Accumulative accuracy of LDA classification after performing 100 training and 100 independent test evaluations for each pip. At each run, the ten random pips from the assemblage were selected as a random training group, and the remaining five pips were classified independently; (**B**) F1 and Kappa **s**cores of random fresh vs fresh data splits with 100 iterations, (**C**) Classification accuracy histogram—100 training [10 samples] and tests [5 samples], gaining mean accuracy of 91%. Cabernet Sauvignon*—1024.

Upon the training set selection, we formed a Mean Square Error (MSE) matrix of size 40 × 40, as the LDA input, by applying an ICP algorithm between every training sample (pip) pair represented as point-cloud. Following the same steps, we formed a test MSE matrix of size 20 × 40 as the test LDA input, where each test sample pip was classified independently. Randomly selected train/test splits allows to decrease any data set bias as well as statistically characterize the impact of the ICP algorithm initialization randomness^41^, i.e., when initiating the comparison between two-point clouds by the ICP, a random point is selected form the target cloud and matched to the closest corresponding points at the source cloud. Figure 2A presents the classification statistics for each variety over the multiple tests.

The number of times a pip was classified at any group is displayed as a yellow bar. If a pip was always classified into the same group, then a single bar is plotted, and it covers the full height of the corresponding group (i.e. 100% classification). However, when classified into several groups, corresponding bars are displayed, and their heights are proportional to the classification’s percentages. The highest classification accuracy of 96% was obtained for Cabernet Sauvignon, the lowest accuracy of classification of 88% was obtained for the 13 variety. In comparison, classification accuracies of 90.4% and 89.6% were accepted for 292 and 9003 varieties, respectively. Figure 2B presents F1 and Kappa **s**cores (maximum of 1) of random fresh vs fresh data splits with 100 iterations, and Fig. 2C presents the total accuracy distribution over all 100 tests, with a mean value of 91% and a standard deviation of 6%.

### Linear discriminant analysis of charred grape pips

**Figure 3:**
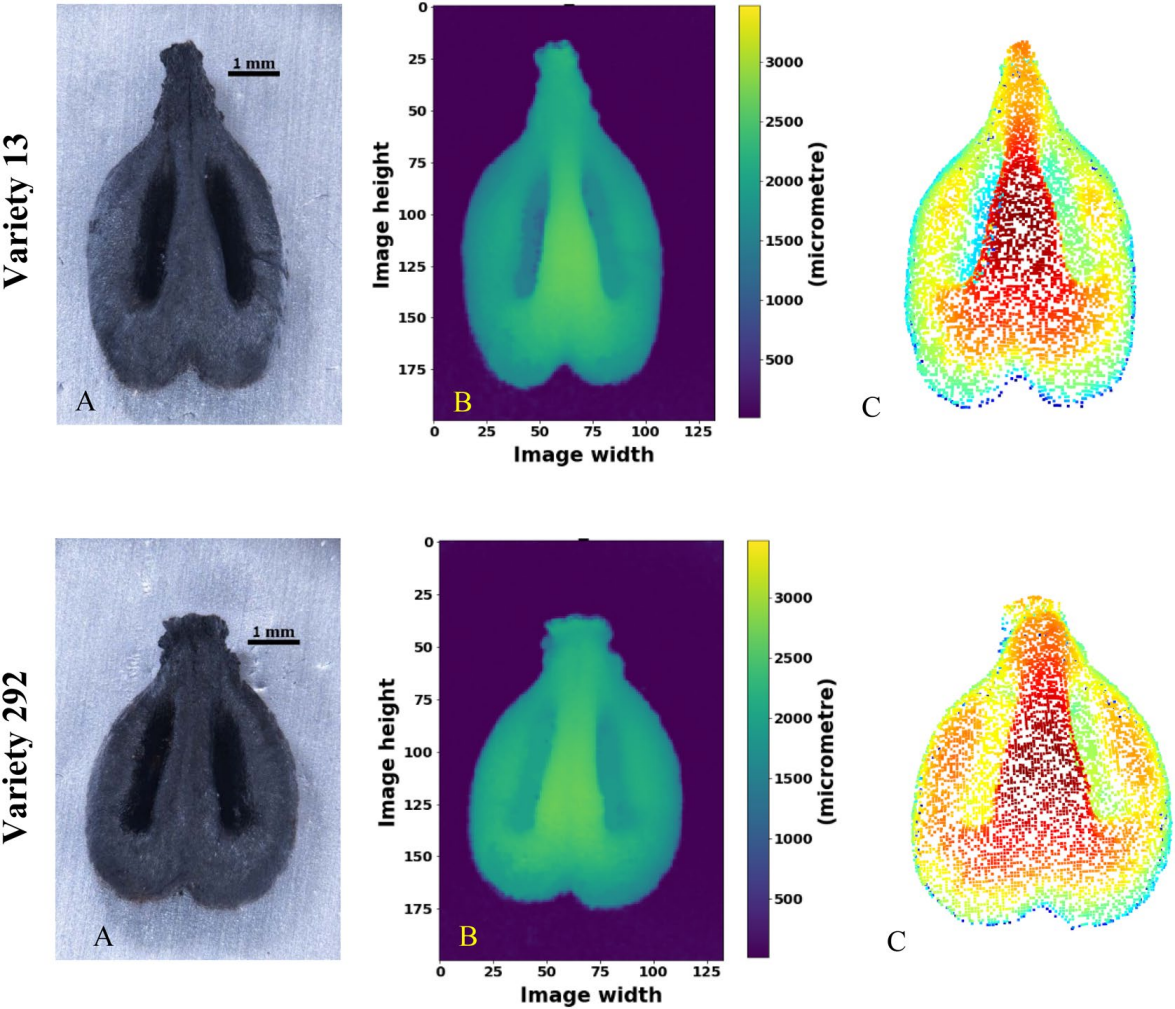
Acquisition of 3D data for charred grape pips. (**A**) High quality focused 3D image; (**B**) height map, and (**C**) points cloud of two varieties of charred pips.

Archaeobotanical seeds are preserved for many years due to charring. Unfortunately, seeds become deformed as a result of exposure to heat^42–44^ and the degree of deformation depends on the charring conditions^45^. Nevertheless, we hypothesized that our suggested 3D morphological classification method might overcome the limitation poised by the deformities and yield good classification results. We started with acquiring high-quality charred seed scans, in which the stereo-microscope proved to be a good selection, as opposed to scanning by a high-resolution 3D scanner—‘PT-M’ (not shown). The resulting high-quality scans enabled transforming the data into cloud point representations, similar to those achieved for fresh seeds (Fig. 3).

**Figure 4:**
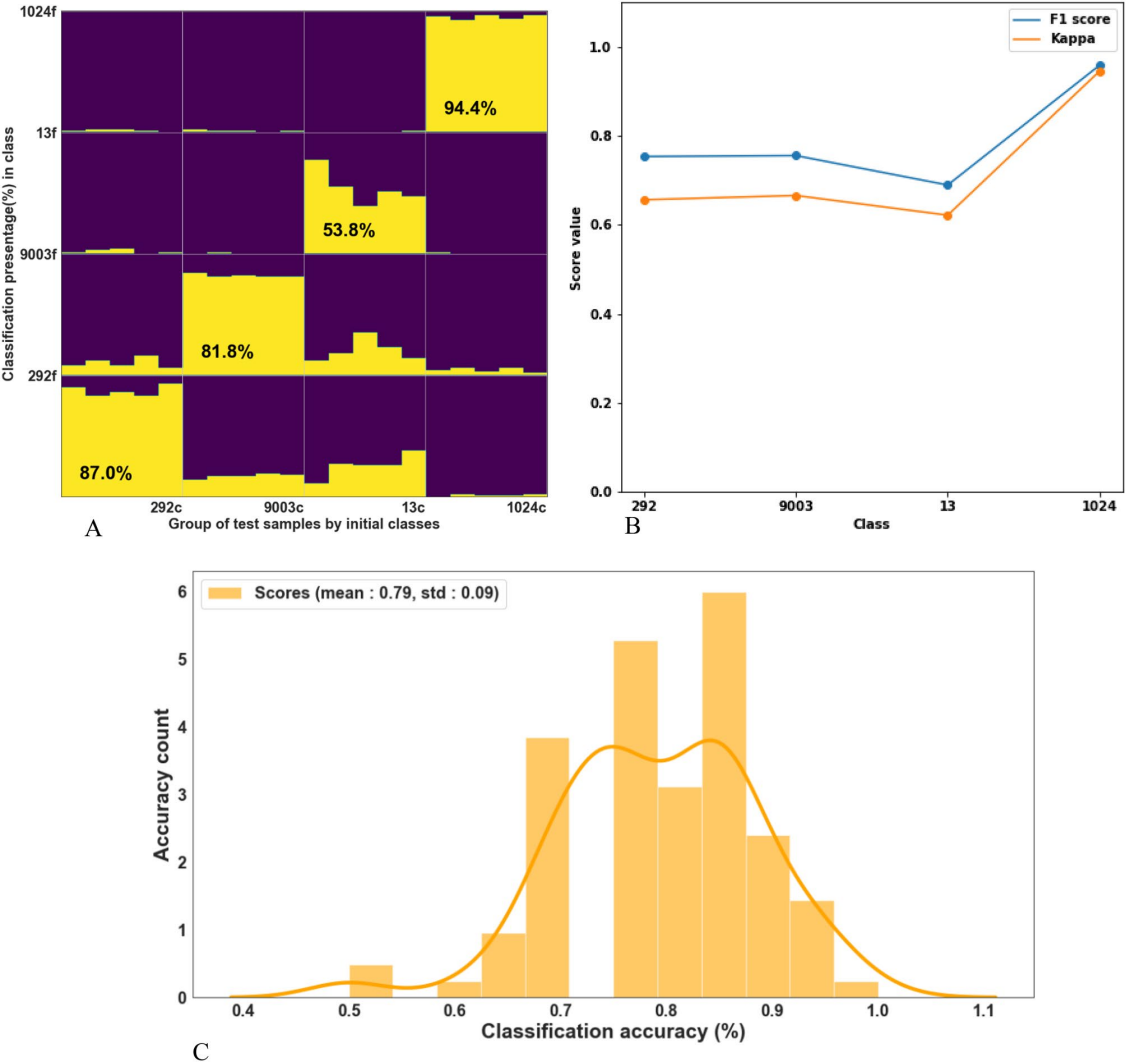
Classification of charred pips using fresh pips as a train set. (**A**) Accumulative distribution of LDA classification after running 100 tests. At each run, 10 random pips from the assemblage were selected as the constant training group, and the 5 remaining burned random pips were classified independently; (**B**) F1 and Kappa scores of random fresh vs charred data splits with 100 iterations, (**C**) Classification accuracy histogram—100 training [10 fresh samples] and independently 100 tests (5 remaining samples).

Hence, we set to evaluate the classification accuracy of charred pips from 4 grape varieties, using 10 fresh pips from each variety (i.e. 40 pips in total), in order to characterize the burning effect. This was done by scanning the fresh pips for training purposes, then charring them, and re-scanning them in charred form for test purposes. As previously explained, 100 consequent evaluations were conducted. Figure 4A presents the classification statistics for each variety. The highest classification accuracy of 94.4% was obtained for Cabernet Sauvignon, while the lowest classification accuracy of 53.8% was obtained for variety 13. Classification accuracies of 87% and 81.8% were obtained for varieties 292 and 9003, respectively. Figure 4B presents F1 and Kappa scores of random fresh vs charred data splits with 100 iterations Fig. 4C represents the distribution of accuracies over all 100 tests, with a mean value of 79% and a standard deviation of 9%.

**Figure 5:**
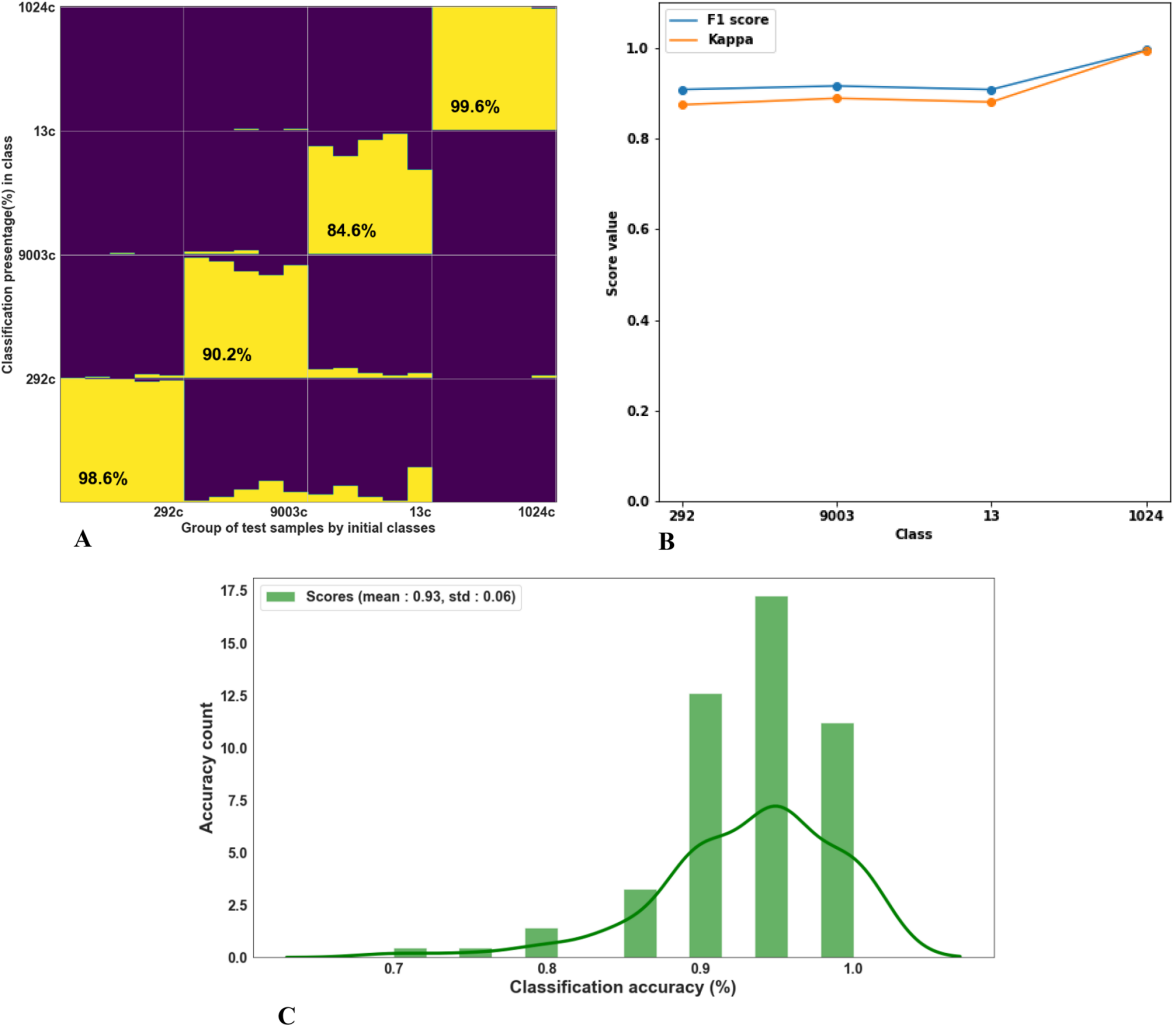
Classification of charred pips using a charred training set. (**A**) Accumulative distribution of LDA classification after running 100 tests. At each run, ten random pips from the assemblage were selected as the training group, and the other remaining five pips were classified, (**B**) F1 and Kappa scores of random charred vs charred data splits with 100 iterations, (**C**) Classification score histogram—100 training [10 burned samples] and independently 100 tests for each pip [5 burned samples].

Finally, we conducted an experiment to assess the classification efficiency when using 10 random charred pips to train the machine, followed by testing unclassified random 5 charred pips originating from the same varieties. This experiment was conducted in order to examine whether we can benefit from training charred pips for classify charred pips. In nature, most of the archaeobotanical specimens found are charred. Therefore, building classification framework which is trained based on charred pips in the first place, can lead to enhanced classification results. Figure 5A presents classification statistics for each variety over 100 separated runs. Very high classification accuracy of 99.6% and 98.6% was received for both Cabernet Sauvignon and variety 292, while varieties 9003 and 13 gained high accuracies of 90.2% and 84.6%, respectively. Figure 5B presents F1 and Kappa scores of random charred vs charred data splits with 100 iterations Fig. 5C represents the distribution of accuracies over all 100 tests, with a mean value of 93% and a standard deviation of 6%.

### Towards the classification of a broader set of varieties

**Figure 6:**
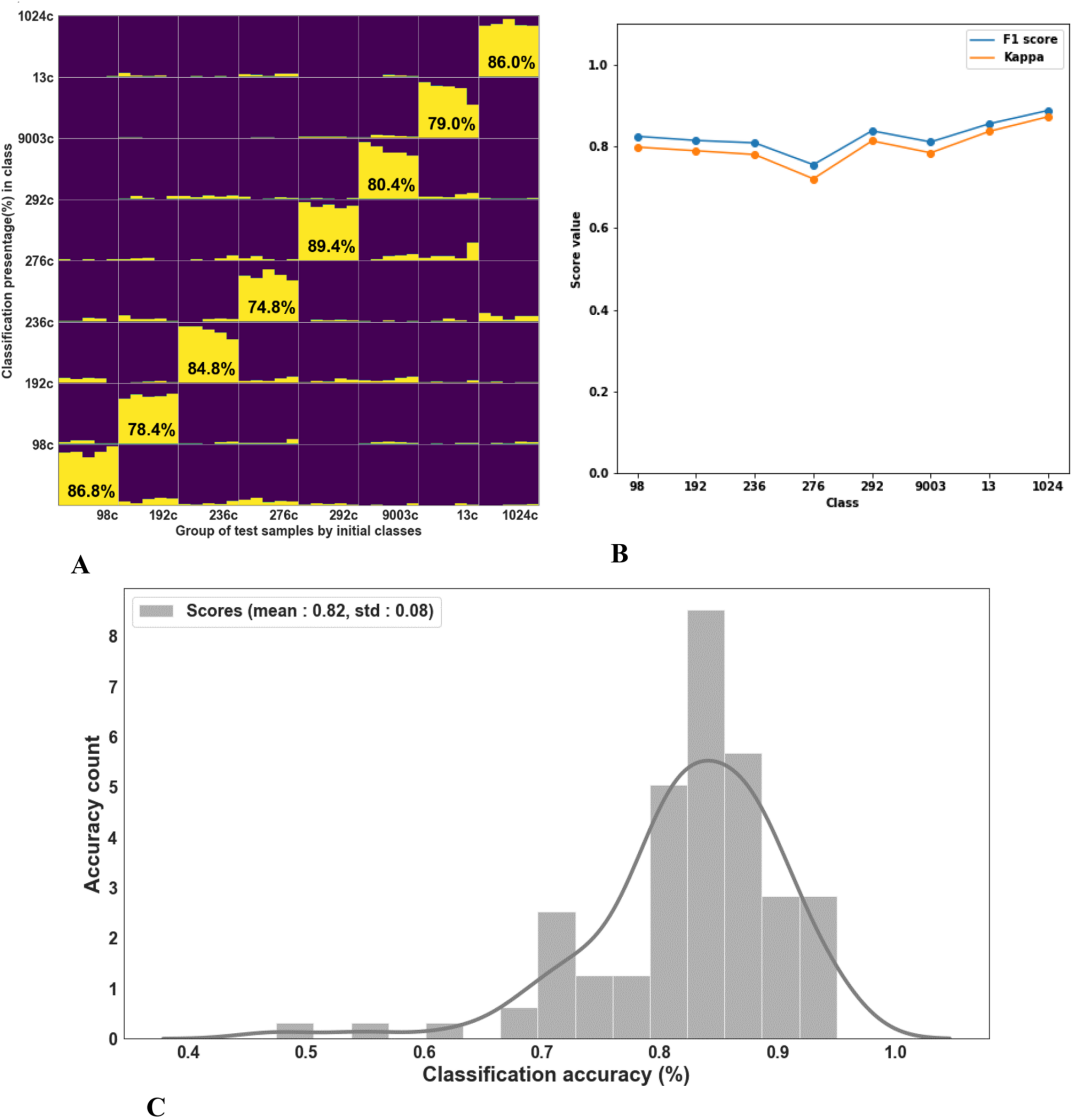
Classification of charred pips from 8 varieties. (**A**) Classification accuracy (y-axis) over 100 iterations on each test sample (x-axis), (**B**) F1 and Kappa **s**cores of random charred vs charred data splits with 100 iterations of 8 varieties, (**C**) Classification accuracy score histogram of 8 varieties—100 training [10 charred samples] and independently 100 tests for each pip [5 charred samples].

In order to examine the scalability of the suggested methodology described above, we performed an additional classification experiment involving eight grape verities. We added four additional charred varieties (N = 15) (98 is *Vitis vinifera* ssp*. sativa* and 192, 236, 276 are *Vitis vinifera* ssp*. sylvestris* lines (wild grapevine) to the four charred varieties used in our previous classifications (292, 9003, 13, 1024). Ten random pips from each variety were chosen as a training group, while five remaining were kept out for testing. In total, we conducted 100 training and classification evaluations of LDA, in which a total of 80 charred samples were used as a train set and 40 charred samples as the test set. Figure 6 shows the classification statistics.

As shown in Fig. 6, the classification accuracy for all varieties was varied between 74.8 and 89.4% %, while the highest classification accuracy of 89.4% was received for variety 292. Compared to the high classification accuracy achieved for four varieties, these results indicate that the classification accuracy degrades when increasing the number of classes. Obviously, to develop a practical method that enables the classification of a seed of unknown origin, the reference population will contain a large number of varieties. As a result, the signal magnitude of each group will be reduced dramatically relative to the entire collection, and no clear learning can be gained based on the training set. Thus, to improve the accuracy for a broader variety set, we suggest implementing a “tournament” classification flow. This innovative approach is based on classifying an unknown seed by conducting multiple LDAs, trained with group sets of a small number of varieties (for example, four). The variety selected from each group are forwarded to a next stage, in which a new group of varieties is trained into group sets of four that are used for the classification of the tested pip. This configuration reduces the number of remaining candidate varieties by a factor of 4 at each stage. Finally, a last group of the highest-ranking candidate varieties will be used as for a final LDA, producing the variety best-fitted to the tested pip, out of the entire tested population (for an illustration of the proposed process, see Fig. 9).

### Testing the classification accuracy of eight varieties by a tournament classification flow

**Figure 7:**
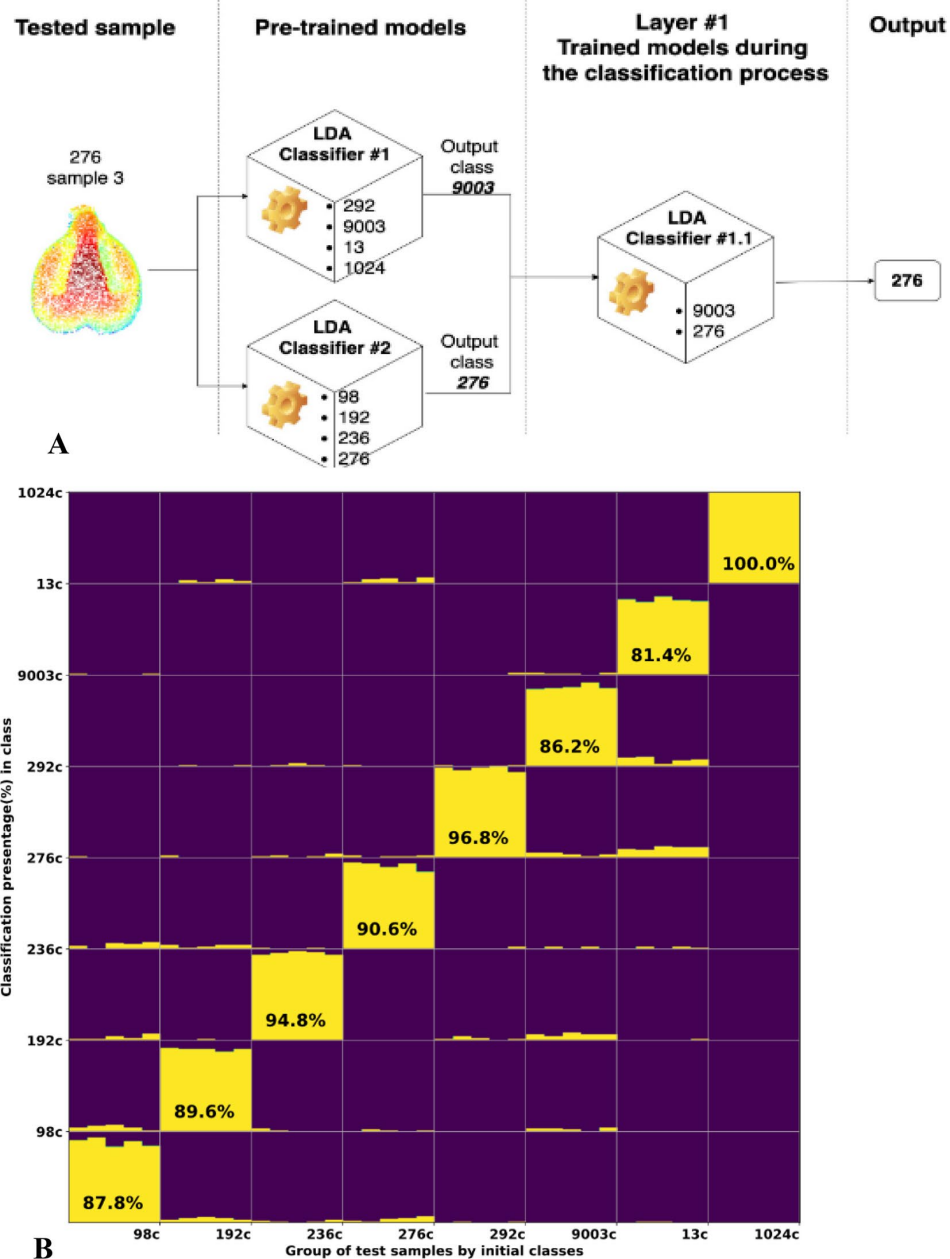
Classification of charred pips from 8 varieties by the “tournament” flow method. (**A**) Tournament classification flow for eight varieties, (**B**) Accumulative distribution (y-axis) of LDA classification of 8 varieties of the “tournament” flow method, after running 100 iterations for each test pip sample (x-axis).

To demonstrate our suggested methodology for classifying a large variety number (in this specific case, eight), we implemented a tournament classification flow experiment, aimed to distinguish between 8 different charred verities, by initially training two individual LDA classifiers (Fig. 7A). Classifier *A* was trained (using ten random pips) to distinguish between varieties 292, 9003, 13, and 1024, as previously described. Additionally, classifier *B* was trained in the same way to distinguish between varieties 98, 192, 236, and 276. The remaining 40 samples (5 from each variety) were kept out of the training as a test group. We then evaluated the classification accuracy of each test sample over 100 iterations in the following way: In the first stage of the tournament, each test sample was classified by classifier *A*, and then by classifier *B.* In the second stage, we trained a new LDA, which aimed to classify the test sample between the two “winning” results, which were selected during the first stage (Fig. 7A). Finally, the selected variety was reported by the LDA of the second stage (see Fig. 7B) as the tested pip’s variety. Figure 7B shows the classification results. All varieties were classified by this method with an accuracy ranging between 81.4 and 100%, with a total average of 90.9%.

**Figure 8:**
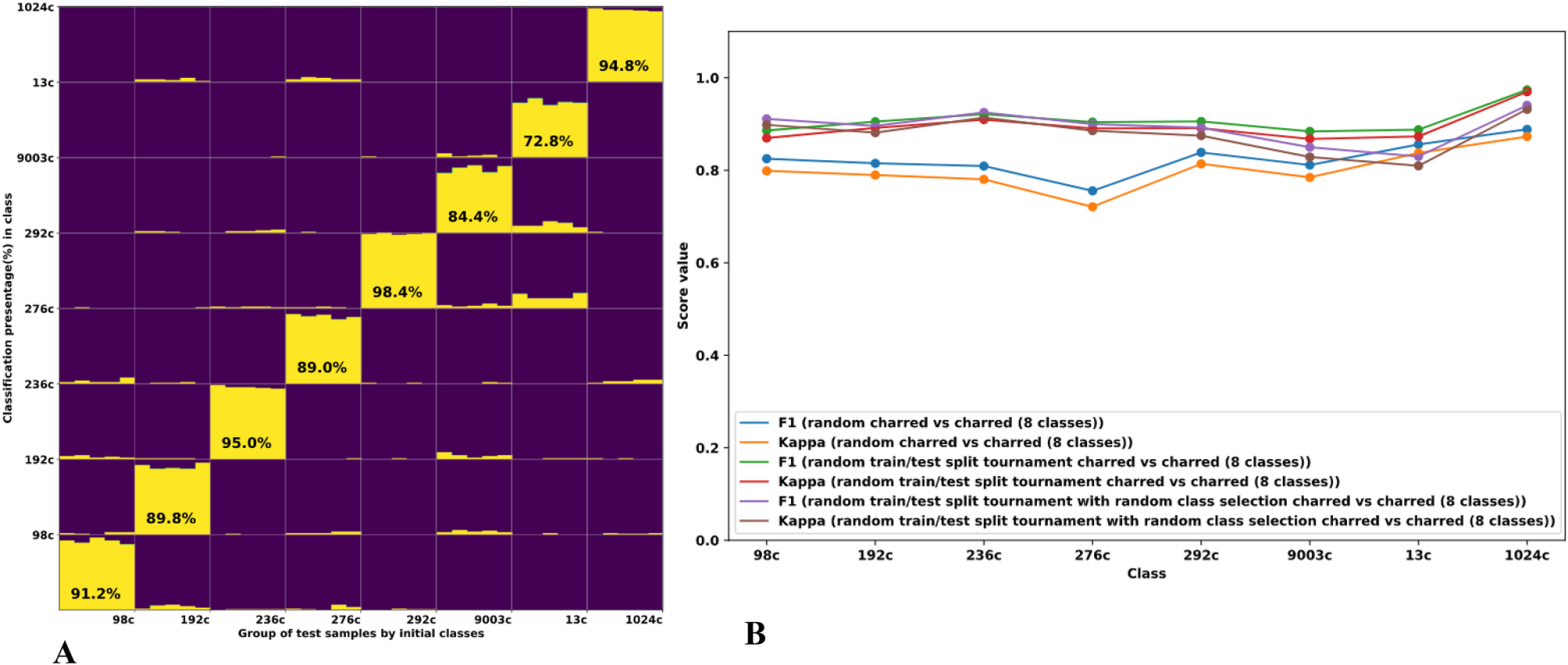
Classification of charred pips from 8 different varieties by applying the “tournament” flow method with random sub model selection. (**A**) Tournament classification statistics charred vs charred 100 test evaluations with random sub model selection, 10 random pips were chosen for test and remaining 5 charred pips for test, (**B**) classification scores of tournament-based methods compared with a non-tournament random charred vs. charred classification with 100 iterations.

In addition, an experiment with random sub model selection was also performed. Similar to the experiment described above, we implemented a tournament classification flow, which aims to distinguish between 8 different charred verities. The main difference from the previous model is that now classifier *A* and classifier *B* were trained (using ten random pips) with random sub model (different 4 varieties) for each iteration to distinguish between varieties 292, 9003, 13, 1024, 98, 192, 236, and 276. Figure 8A presents the classification results. The classification accuracies obtained for several varieties were slightly lower than the classification accuracies obtained from the previous experiment (see Fig. 8A). Figure 8B presents a comparison of classification scores between all 3 methods used for classifying the 8 different varieties. It is shown that the random train/test split tournament with or without random class selection yield higher F1 and Kappa scores compared with the non-tournament method (random charred vs. charred).

## Discussion

In this work, we demonstrated a successful varietal classification of charred and fresh grape seeds using an accessible 3D scanning method. Various grape pips positions, plate surfaces materials, and stereoscopic focus intervals were tested to achieve optimal conditions for accurate scanning. We used a Nikon SMZ25 stereo microscope to create scan sets of grape pips and designed the conversion of the resulting “wrl” files into a cloud point representation. An automated stereoscopic microscope is an available tool found in many labs, making our method of using the full data set gained by 3D data for separating the varieties by their morphological trait, available and approachable. This new approach is the next step in the journey for grape variety identification for agriculture and archaeobotanical purposes, started by the development of the traditional morphometric methods and the elliptic Fourier transform method^16–18,46^ later applied for charred and archaeological findings by analysis of surface morphology^45,47–51^. Our study utilizes the LDA algorithm classification advantages for classifying preprocessed stereoscopic grape seed images. Our meticulously tailored preprocessing phase constitutes data representation that emphasizes the morphological distance between seeds type, based on a cloud point data structure. We define such distance as the minimum Mean Square Error (MSE) of the Euclidean distance between two cloud points measured by the Iterative Closest Point (ICP) algorithm. Our method discriminates between and accounts for three types of errors (the error reduction method is discussed later): (1) Human error—positioning of each sample by hand may introduce a lack of homogenous scanning, (2) ICP algorithm converge error—Applying ICP from sample A to B might be different from applying it from B to A due to the cost function defined in the ICP algorithm, and (3) The ICP algorithm introduces randomness, i.e., using ICP from A to B might differ in every iteration. Concerning the above mentioned possible introduced errors, we implemented the following steps: (1) We developed a scanning protocol while designing an alignment technique that reduces human error possibility, (2) We forced the ICP algorithm to include all data points in a cloud data structure representation to calculate minimum distance; such constrain minimizes the difference between the two way ICP evaluation, and thus emphasizes morphological similarities, and (3) We construct a proper statistical analysis, based on a vast amount of evaluations to examine our classification accuracy distribution under the suggested methodology. In addition to the errors mentioned above, which could lead to possible misclassifications, trivial biological causes such as natural deformations can also lead to misclassification of seeds, as shown in Fig. 4A for class 13C.

Furthermore, our suggested classification approach presents many advantages, which enhances its applicability for variety identification by seed, as compared with similar works. For example, Karasic et al.^38^ used Fourier transform coefficients as a Machine Learning input and perform PCA for clustering visualization applied with 3D pip scans, which demand a meticulous scanning process and high accuracy of positioning. Bouby et al.^46^ used Elliptical Fourier Transform (EFT) to extract dominant features from dorsal and lateral image outlines utilizing LDA for classification. The last study presented the results based on the “leave-one-out” folding method with posterior classification (P > 0.75). The "leave-one-out" statistical analysis is informative for one sample classification, as each iteration test-error is unbiased, but makes it difficult to generalize the model's ability to classify the entire given group-set, such as it has a high variability as only one observation validation-set is used for prediction. In addition, both studies utilize EFT which is sensitive to scanned samples placement as described by Haines and Crampton^52^ and was also noted by Karasic et al.^38^: “A crucial step before any comparison of shapes is to have a robust and system- atic method of positioning the object that enables precise and repeatable measurements”. In contrast, our suggested scanning method is less sensitive to inaccurate positioning of seeds; this simplifies and speeds-up data acquisition. Additionally, our results indicate good classification accuracy along with adequate ML model data generalization, given the selected representative sets. This was partially achieved since the ICP methodology increases the homogeneity in feature comparison between two given samples due to its algorithm nature. Thus, our proposed method might be suggested as suitable for big data analysis.

Implementing our methodology for classifying fresh and charred grape seeds of four varieties, we achieved a mean accuracy level of 79% for fresh (train) vs. charred (test) pips, mean accuracy of 91% for fresh vs. fresh pips, and unpredictably, the highest classification rate of 93% was achieved when charred pips were used as a training set to identify charred pips. These results emphasize that burned samples show increased morphological similarity compared to fresh ones, possibly due to the removal of various fresh soft tissues present on the seeds surface, which reduces “structural noise”.

These results also indicate that a future methodology for archaeobotanical remains identification might be best implemented on the construction of a reference database of a large set of charred grape pips of the populations of reference varieties, for the accurate classification of unknown archaeological charred grape pips. The applicability to other important plant species is yet to be determined, as the deformities caused by charring may differ between species. In addition, the relatively high classification rate achieved when using a fresh seeds training set to classify a charred test set suggests that although typical morphological changes occur in grape pips following charring^42,43,45,46^, our 3D identification approach can overcome this barrier, and suggests that identification results may also be achieved by using fresh pips reference data set towards possible identification of charred seeds, without the need for an empirically calculated charring compensation key.

Although the ICP based morphological classification method shows comparable and distinguishable results, the need for a more robust and deterministic algorithm still exists. One way towards such improvement is to take advantage of the scanned sample rigid structures, represented as a 3D surface. Such representation captures spatial morphological features, in contrast to cloud points representation based on discrete Euclidean points. Several previous studies attempted to find different metrics for surface similarities. A most recent work by Lipman at el.^53^ used conformal mapping to map 3D scanned teeth of mammals, found on an archeological site, to a unit disk probability space, and defined the Wasserstein distance as the metric between them. Utilizing Differential Geometry in future works can be an essential key for building a robust, efficient, and accurate classifier that can handle hundreds and thousands of grape varieties.

**Figure 9:**
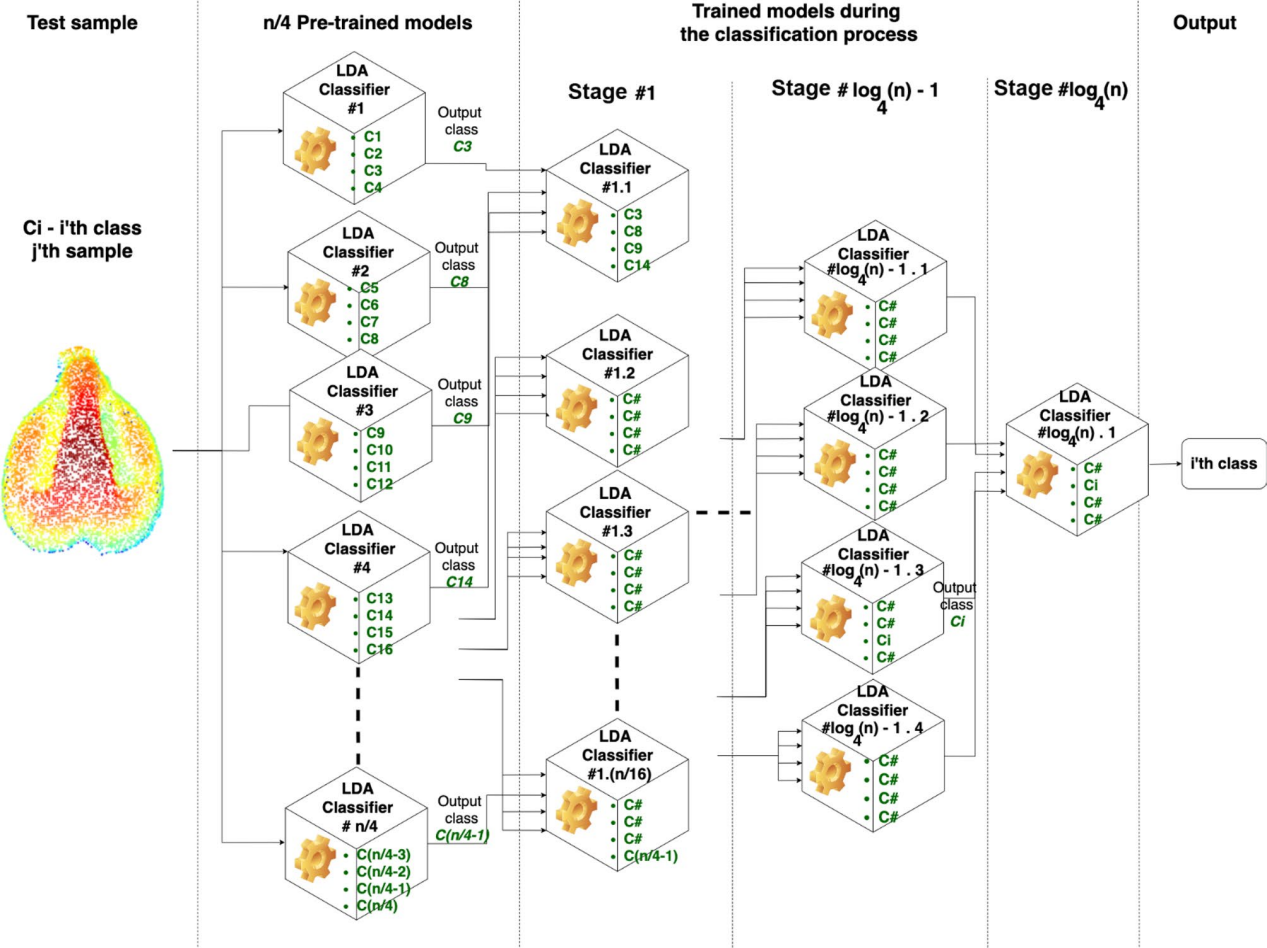
General demonstration of a “tournament” classification flow and its internal stages of training and classifications down to the final identification result. The test sample may be an unidentified charred grape pip, recent or archaeological. The whole reference population, which is believed to include the tested sample’s variety, is divided into random groups of four varieties, trained as separated machines. At the end of the first step, the selected variety of each machine will be elevated to the next stage, where again, the newly established set will be randomly divided into groups of four varieties, and so on, until the final stage will recognize the most probable identification result.

Our method, which demonstrates promising results, still requires handling the need for identification of an unidentified pip against a broad set of reference varieties. Currently, classification results are dramatically better when a small group of varieties is classified at one point, dropping with the addition of varieties. We propose to address this issue by either implementing the suggested “tournament” methodology analysis (see Fig. 9), in which the seed is identified by classification against pre-trained and “on flow” trained multiple models, such that each model will be trained to distinguish between four different classes; the classification flow will be divided into ${{\log}}_{4}n$ stages, where n denotes the number of varieties. As a first stage, the sample will be classified by n/4 pre-trained classifiers. Then, in every following step, a new LDAs will be trained based on the output of the previous stage and trained only with the initial training subsets. As a final stage, a four varieties (or less) LDA classifier will be trained and output the final result. We recommend evaluating each sample with at least ten iterations in order to gain reliable statistics. Figure 9 shows the general case of such implementation.

In addition, we suggest that future approaches will utilize advanced ML algorithms such as Deep Learning (DL). Nevertheless, this approach requires a vast amount of training samples for each verity, which will demand an even more robust and less time-consuming scanning method. We are currently exploring the mentioned above approaches towards implementation in the identification of charred archaeological grape seeds.

## Conclusions

The presented innovative 3D classification method shows good classification results for fresh grape seeds and even higher accuracy levels for charred ones. To our knowledge, this is the first application of a 3D classification tool which makes use of the full 3D data set. This tool can be further developed to accurately identify charred archeological seeds, which may present a breakthrough in any taxonomy-related field.

## Materials and methods

### Plant material

A total of 60 seeds from 8 cultivars were sampled. Grapes from the endogenous Israeli varieties 9003 (Dabuki M.), 13 (Marawi), 292 (Tzuriman S.), 98 (Homra Pisga), 192 (Banias 1), 236 (Samach Harduf), and 276 (Banias Shaar) were collected from the endogenous varieties vineyard collection^39,40^ or in the wild and Cabernet Sauvignon grapes were collected from the European varieties collection vineyard, Israel. Mature seeds were extracted from ripened grapes, collected from at least three different grapevines. The seeds were washed by water to discard any residual pulp tissue and air-dried for two days, then stored in a closed vial until used. Before scanning, each seed was carefully cleaned by brushes and needles from any external tissues coating its crevices, to enable an effective scan of the seeds’ topography. This was done following experiments showing that a scan of uncleaned seeds does not capture a substantial part of their structure (sup. Fig. 1). Fifteen seeds were prepared from each variety for the scans.

### Imaging by stereo microscope

An aluminum foil was chosen as the optimal surface for accurate scan results, out of various tested surfaces: white paper, black paper, glasses with different coatings, etc. Grape seeds were placed on a microscope glass slide wrapped with aluminum foil, set under the microscope and illuminated with LED spots. A polylactide (PLA) light cap was designed and printed using ULTIMAKER to achieve a uniform and equal luminescence, needed for high-quality scans. Four LED spots were glued inside the self-designed cap (sup. Fig. 2). Images were taken with a Nikon SMZ25 stereo-microscope (Nikon, Tokyo, Japan) equipped with a Nikon DS-Ri2 microscope camera. Sixty digital micrographs (resolution: 4908 × 3264 pixels), with each step about 50 µm, were taken at different focal planes and compiled to a single image using ND2-NIS elements software with an Extended Depth of Focus (EDF) patch (Nikon Instruments, Japan). To construct a 3D image scan of the seeds, sequential imaging of the ventral side positioned horizontally was conducted by the stereo microscope, with intervals of approximately 50 µm. These images were then transformed into a single high quality focused image in a “wrl” file format using the dedicated stereo microscope’s software.

### Heating experiment

Fresh grape pips were heat-treated to study the impact of charring on morphological changes in pips. For this purpose, clean grape pips were scanned by a stereo-microscope before and after charring. Pips were heated in batches of 15, at a temperature of 230 °C for 2 h under low oxygen availability (covered with a thick layer of sea sand) to prevent the burning of the pips^42,44,45,54^.

### Data preparation

Given a scanned data sample in the “wrl” file format, the first step is converting it to a cloud point representation (see Fig. 1, Bottom). The conversion was achieved by multiplying every coordinate (${\overrightarrow{c}}_{i}$).

$${\overrightarrow{c}}_{i}=\left({x}_{i},{y}_{i}\right)\in F$$

Inside the “wrl” file ($$F)$$, excluding coordinates that represent the surface plate itself, by the inverted intrinsic matrix ($${K}^{-1}$$).

$${K}_{3x3}=\left[\begin{array}{ccc}{f}_{x}& s& {c}_{x}\\ 0& {f}_{y}& {c}_{y}\\ 0& 0& 1\end{array}\right]$$

The intrinsic matrix (K) contains parameters that describe the visual sensor characteristics: $({f}_{x},{f}_{y})$ are the focal-lengths, $\left({c}_{x},{c}_{y}\right)$ are the sensors center coordinates in pixels and $\left(s\right)$ is the skew parameter^55^. Then, the coordinate (${\overrightarrow{c}}_{i}$) is scaled by its corresponding height map value (${z}_{i}\in F$—z-axis coordinate).

$$\begin{aligned} {\overrightarrow V _{{c_i}}} & = {K^{ - 1}} \cdot {z_i} \cdot \left[ {\begin{array}{ll} {{c_i}}&1 \end{array}} \right] \\ & = {K^{ - 1}} \cdot {z_i} \cdot \left[ {\begin{array}{lll} {{x_i}}&{{y_i}}&1 \end{array}} \right] \end{aligned}$$

Intrinsic matrix parameters were determined manually based on the actual surface dimensions of the scanned image sample, along with the distance between the surface and the visual sensor. As a next step, we defined the training and test sets size in the following way: 10 random variety representative pips as the training sets and the remaining five pips as the test sets for multi-class classification scenario and for the heating classification scenario. The training set is described as an M by M matrix, where M is the total number of training samples (i.e., ten pips from each variety for the multi-class classification and for the heating classification). The matrix is constructed such that every $i,j$ matrix entry represents the Mean Square Error (MSE) of the applied Iterative Closest Point (ICP) algorithm on points-cloud sample $i$ and points-cloud sample $j$ (i.e., for the multi-class classification scenario and for the heating classification scenario with four different varieties, we get 40 by 40 matrix.The test set matrix was constructed in the same manner as the training set, except that in this case, the test matrix size has N by M dimensions, where N is the total number of test samples (i.e., N = 20 = 5 * 4 for the multi-class classification scenario, and for the heating classification), and M is the total number of training samples (i.e., M = 40 = 10 * 4 the multi-class classification scenario, and fresh (uncharred) pips for the heating classification). An example of the training and test matrix representation for the multi-class classification scenario is shown in sup. Fig. 3.

For the case of classifying individual pip, the test matrix becomes a single vector with a size of 1 by M. As such, for different classification scenarios (multi-class classification and heating classification), a different LDA model was trained based on the scenario training matrix (set) as an input. Each LDA model was evaluated based on the test matrix (set) as an input according to the classification scenario. We note that the evaluation of a test pip set is equivalent for evaluating individual pips one by one since the test pips are treated independently in the test set matrix.

### LDA analysis

Linear Discriminant Analysis (LDA) is a supervised machine learning technique used for classification problems^56^. Similar to PCA, LDA uses dimensionality reduction as a preprocessing step, but in contrast to PCA, LDA considers data labels. The LDA method creates a projection of high dimension features onto low dimensional space in three necessary steps:

1. It calculates the separability between different classes.
  $${S}_{b}=\sum _{i=1}^{g}{N}_{i}({\bar{x}}_{i}-\bar{x})({\bar{x}}_{i}-\bar{x}{)}^{T}$$
2. It calculates the distance between the mean–variance of each class (“within-class variance”).
  $${S}_{w}=\sum _{i=1}^{g}{(N}_{i}-1){S}_{i}=\sum _{i=1}^{g}\sum _{j=1}^{{N}_{i}}({x}_{i,j}-{\bar{x}}_{i})({x}_{i,j}-{\bar{x}}_{i}{)}^{T}$$
3. It constructs a low-dimensional space such that it maximizes the mean–variance for each class (“between class variance”) and minimizes the mean–variance between different classes (“within-class variance). Let P be lower dimensional space projection, which is called Fisher’s criterion.
  $${P}_{ida}={\arg}{\mathit{max}}_{P}\frac{\left|{P}^{T}{S}_{B}P\right|}{\left|{P}^{T}{S}_{w}P\right|}$$

Furthermore, we used the “discriminant_analysis” python package from the “sklearn” library to train and evaluate the LDA performance [https://scikit-learn.org/stable/modules/generated/sklearn.discriminant_analysis.LinearDiscriminantAnalysis.html]. The train set matrix in the LDA were trained on its default values and then assessed on the test set matrix.

### ICP

Iterative Closest Point (ICP) is a registration algorithm designed to find a transformation between two given point clouds that minimizes any arbitrary objective function^57–60^. We used the Open3D library to perform ICP on the samples. [Open3D: A Modern Library for 3D Data Processing].

### Metrics

Every conducted experiment was evaluated with three different scores: accuracy, F1 and Kappa. The accuracy metric describes the ratio between correct classifications and the total number of classifications as percentage. The F1 score conveys the balance between the precision and the recall and was calculated using sklearn python library (“sklearn.metrics.f1_scor”). The Kappa score represents the extent to which the data collected in the study are correct representations of the variables measured, and was calculated using skelarn python library (sklearn.metrics.cohen_kappa_score).

### Tournament

The tournament methods are evaluated in two ways: the first one utilized random selection of train and test samples split for each iteration. The second experiment also utilized random train and test samples split for each iteration, but with the addition of random selection of the groups upon the LDA machine which were trained at each iteration. This was done in order to compare all three methods for classifying the 8 different class, and infer whether there are any differences between them. For the general case (deployment model), the tournament will operate by pre-defined groups at every tournament layer and pre-defined training set.

### Plant material statement

Experimental research and field studies on plants comply with relevant institutional, national, and international guidelines and legislation. The plant material (seeds) was collected either in the wild in Israel, according to permit 41958 initiated by the Israel Nature and Parks Authority (for the wild varieties) or from the collection vineyard managed by the authors at Ariel.

## Supplementary Information

Supplementary Information.

## Data Availability

The data supporting the findings of this study are available from the corresponding authors.
