# Supplementary material for: Accurate classification of fresh and charred grape seeds to the varietal level, using machine learning based classification method
Source: Sci Rep. 2021 Jun 30;11:13577. doi: 10.1038/s41598-021-92559-4 (PMC8245476; doi:10.1038/s41598-021-92559-4)

**Supplementary Information**

**Utilizing machine-learning-based 3D image analysis for classifying charred grape seeds to the varietal level**

Vlad Landa^1^, Yekaterina Shapira^2^, Michal David^3^, Avshalom Karasik^4^, Ehud Weiss^3*^, Yuval Reuveni^5,6*^ and Elyashiv Drori^2,7*^

**Supplementary Fig. 1: 3D images of fresh and charred grape pips of variety 9003. A. Uncleaned fresh pip, B. Charred uncleaned grape pip. C. Cleaned fresh pip. D. Charred cleaned grape pip. E. Charred Uncleaned archaeological grape pip found in a winepress at Beit El, Israel.**


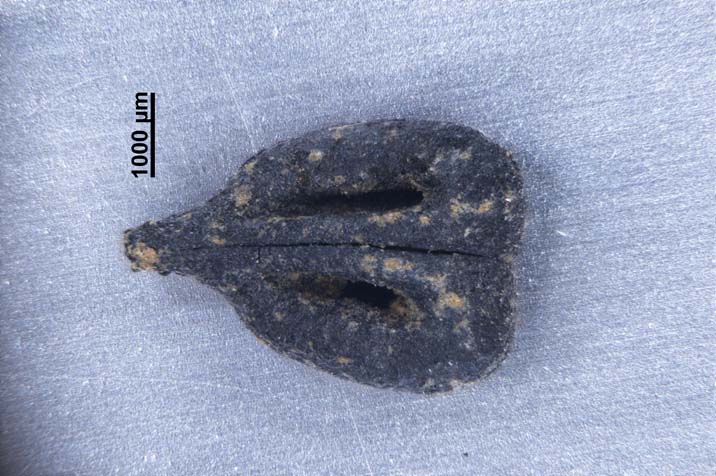

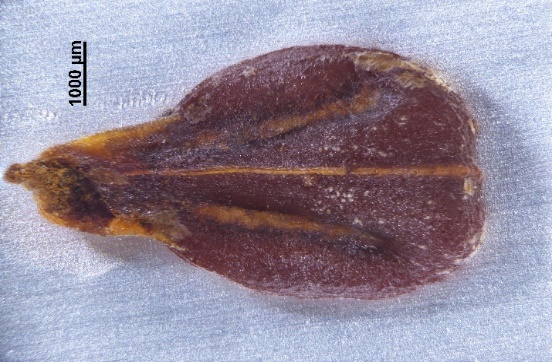

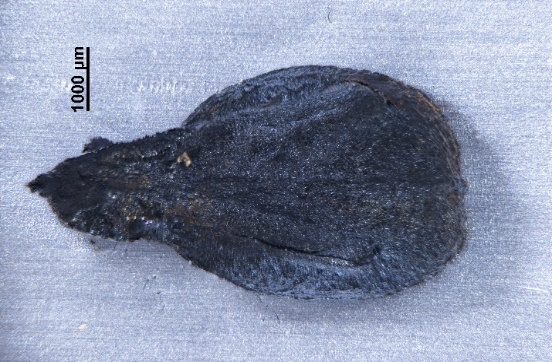

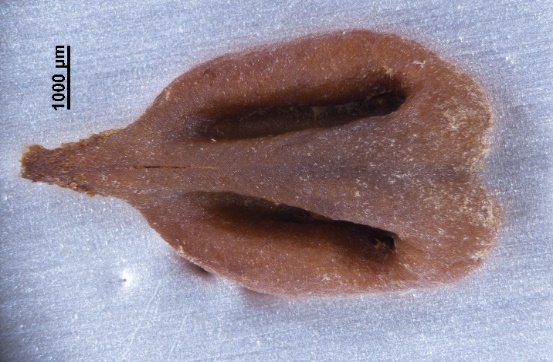

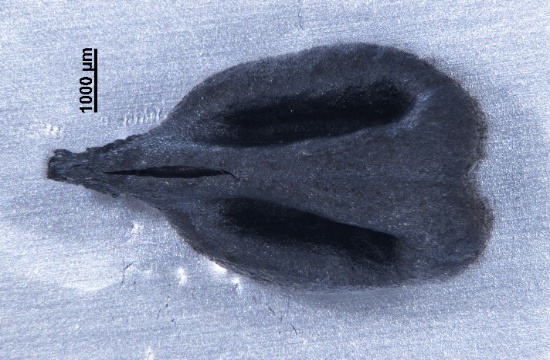


**A**

**B**

**D**

**E**

**C**

Supplementary Fig. 2: PLA 3D printed light-cap: A – front view; B - top view and C – bottom view with four lamp stripes (mm).


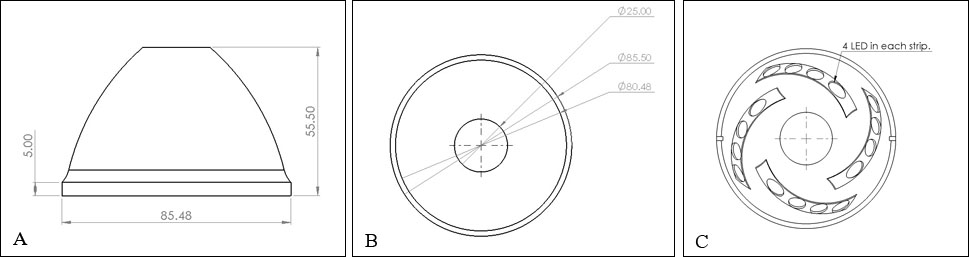


Supplementary Fig. 3: Train and test matrices (sets), each index inside the matrices represents the MSE value reported by the ICP algorithm between pip points cloud $\boldsymbol{i}$ and pip point cloud $\boldsymbol{j}$. A-Train matrix (set) of ICP scores with 40x40 dimensions, 10 constant representative pips (y-axis and x-axis) from each class of 292f, 9003f, 13f and 1024f classes. B - Test matrix (set) of ICP scores with 20x40 dimensions, 5 remaining pips (y-axis) from each class of 292f, 9003f, 13f and 1024f classes and the 10 constant representative pips (x-axis).

A

B


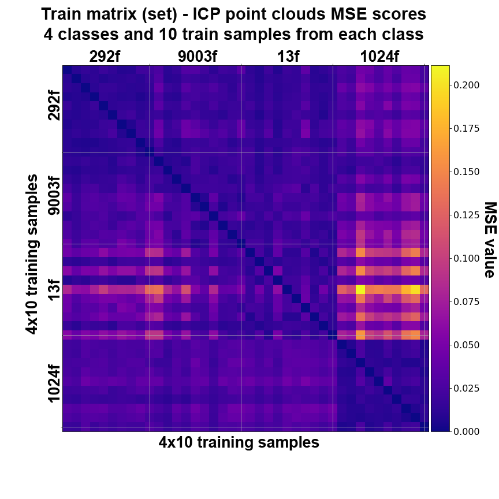

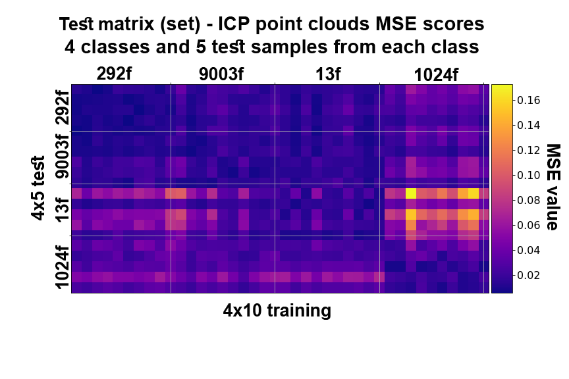

Supplement: Supplementary file 1 — Supplementary Information. [file 41598_2021_92559_MOESM1_ESM.docx]
